# Brain-Derived Neurotrophic Factor/FK506-Binding Protein 5 Genotype by Childhood Trauma Interactions Do Not Impact on Hippocampal Volume and Cognitive Performance

**DOI:** 10.1371/journal.pone.0092722

**Published:** 2014-03-21

**Authors:** Dennis Hernaus, Ruud van Winkel, Ed Gronenschild, Petra Habets, Gunter Kenis, Machteld Marcelis, Jim van Os, Inez Myin-Germeys, Dina Collip, for Genetic Risk and Outcome in Psychosis (G.R.O.U.P.)

**Affiliations:** 1 Department of Psychiatry and Psychology, South Limburg Mental Health Research and Teaching Network, EURON, School for Mental Health and NeuroScience MHeNS Maastricht University, Maastricht, The Netherlands; 2 University Psychiatric Centre Catholic University Leuven, Kortenberg, Belgium; 3 King's College London, King's Health Partners, Department of Psychosis Studies, Institute of Psychiatry, London, United Kingdom; University of Iowa Hospitals & Clinics, United States of America

## Abstract

In the development of psychotic symptoms, environmental and genetic factors may both play a role. The reported association between childhood trauma and psychotic symptoms could therefore be moderated by single nucleotide polymorphisms (SNPs) associated with the stress response, such as FK506-binding protein 5 (FKBP5) and brain-derived neurotrophic factor (BDNF). Recent studies investigating childhood trauma by SNP interactions have inconsistently found the hippocampus to be a potential target underlying these interactions. Therefore, more detailed modelling of these effects, using appropriate covariates, is required. We examined whether BDNF/FKBP5 and childhood trauma interactions affected two proxies of hippocampal integrity: (i) hippocampal volume and (ii) cognitive performance on a block design (BD) and delayed auditory verbal task (AVLT). We also investigated whether the putative interaction was different for patients with a psychotic disorder (n = 89) compared to their non-psychotic siblings (n = 95), in order to elicit possible group-specific protective/vulnerability effects. SNPs were rs9296158, rs4713916, rs992105, rs3800373 (FKBP5) and rs6265 (BDNF). In the combined sample, no BDNF/FKBP5 by childhood trauma interactions were apparent for either outcome, and BDNF/FKBP5 by childhood trauma interactions were not different for patients and siblings. The omission of drug use and alcohol consumption sometimes yielded false positives, greatly affected explained error and influenced p-values. The consistent absence of any significant BDNF/FKBP5 by childhood trauma interactions on assessments of hippocampal integrity suggests that the effect of these interactions on psychotic symptoms is not mediated by hippocampal integrity. The importance of appropriate statistical designs and inclusion of relevant covariates should be carefully considered.

## Introduction

A history of childhood trauma (CT) is prevalent in individuals suffering from psychotic symptoms [1], [2]. The observation that cessation of CT is associated with reduced psychotic symptoms [2] provides further speculation for a link between the two. Although recent studies have attempted to investigate interplay between CT and genetic variation in relation to psychotic symptoms later in life [3], [4], the underlying neurobiology mediating these effects remains poorly understood [5].

Exposure to CT/abuse has been reported to affect hypothalamus-pituitary-adrenal axis (HPA) function later in life: changes in cortisol [6] and adrenocorticotropin hormone [6], [7] release have been observed. At the structural level, the hippocampus has been shown to be particularly sensitive to stress hormone exposure, supported by reductions in neurogenesis [8] and increased atrophy [9]. This finding goes hand in hand with reports of hippocampal volume being decreased in some, but not all, clinical [10] and non-clinical [11] samples that experienced CT. The underlying mechanism behind this decrease may be that significant stress triggers an increase in levels of circulating glucocorticoids [9]. Excessive levels of glucocorticoids negatively affect dendritic branching [12] and neurogenesis [13] specifically in the hippocampus [9]. Given that hippocampal-dependent memory systems develop relatively late [14], this may have deleterious effects on hippocampal-dependent memory maturation and underlying functions later in life.

FK506-binding protein 5 (FKBP5) gene [4] and brain-derived neurotrophic factor (BDNF) messenger ribonucleic acid (mRNA) [3] are expressed in the hippocampus [15], [16], impact on hippocampal functioning [17] and are associated with the stress response [4], [18], [19], suggesting that genetic variation in FKBP5 and BDNF may affect hippocampal morphology and function, potentially contributing to differential sensitivity to CT between individuals.

Studies that have investigated the effect of either CT [10], [11], [20], [21] or genetic variation [40] on hippocampal volume have produced mixed results and were unable to affirm an effect respectively. Furthermore, recent inconsistencies between studies investigating BDNF genetic variance by CT interactions on hippocampal volume [22], [23] make it difficult to interpret the effects of these interactions on the hippocampus, if any it all, and therefore warrant further investigation. These inconsistent results may be related to inappropriate use of covariates in Gene times Environment (G×E) studies [24]: whereas G×E studies should include relevant covariates, the covariate × environment (E) and covariate × gene (G) interactions are often left out of the model. Although these G×E are expected to be subtle, CT by genetic variance interactions on hippocampal volume in the context of psychosis could result in valuable insights into stress-sensitivity.

To date, two studies have investigated the effect of BDNF/FKBP5 by CT interactions on psychotic symptoms in adulthood [3], [4]. These studies investigated single nucleotide polymorphisms (SNPs) with functional properties within the FKBP5 [4] and BDNF gene [3] associated with glucocorticoid receptor sensitivity [18] and neurogenesis/neuroplasticity respectively [25], [26]. Collip and colleagues [4] showed in a general population sample that minor alleles of FKBP5 SNPs rs9296158 and rs4713916, in combination with exposure to CT, were associated with increased levels of psychotic symptoms and blunted cortisol levels in adulthood. These FKBP5 genotype by trauma interactions were also found in different follow-up samples at different levels of psychosis severity and familial liability, although not always consistently so [4]. A recent study [27] demonstrated that the risk for post-traumatic stress disorder is associated with FKBP5 genotype-specific CT-dependent demethylation, in support of FKBP5 genotype by CT interactions in the development of stress-related disorders later in life. Alemany and colleagues [3] showed that the expression of subclinical psychosis in a general population sample was dependent on BDNF Val66Met genotype in those exposed to CT, a finding that remains to be replicated in independent samples [5]. So far, the effect of BDNF/FKBP5 by CT interactions on hippocampal volume in the context of psychosis has not been investigated. Given the presence of such interactions on the behavioural (i.e. symptom level) [3], [4], such interactions might indicate subtle changes in hippocampal structure or function in psychosis.

The present study investigated whether synergistic effects of CT and BDNF/FKBP5 genotype influenced two proxies of hippocampal integrity in a large sample of individuals with psychotic disorder and siblings. These proxies were: (i) assessments of hippocampal volume and (ii) delayed performance on an auditory verbal learning task and performance on a spatial memory task, cognitive tasks consistently demonstrated to be dependent on hippocampal function [28]–[30]. Decreased hippocampal integrity, negative changes in the structure's function, would potentially be reflected in attenuated volume and decreased cognitive performance.

We first investigated the role of BDNF and FKBP5 in the association between CT and hippocampal volume/cognitive performance in adulthood, regardless of illness status, further investigating recent mixed results on the role of the BDNF gene in the association between childhood adversity and hippocampal volume [22], [23]. Given that not all individuals who experience CT develop a psychotic disorder, we further investigated whether the effect of BDNF/FKBP5 SNP genotype on the association between CT and hippocampal volume/cognitive performance was different for patients and their siblings, in order to elicit possible group-specific protective/vulnerability effects. Covariate × E and G were included in all relevant analyses. Model fit tests were performed and covariates associated with hippocampal volume were included.

## Methods

### Sample

All data described in this manuscript pertain to a longitudinal magnetic resonance imaging (MRI) study in Maastricht, the Netherlands [31]). Data from 89 patients with a psychotic disorder and 95 healthy non-psychotic siblings of patients with a psychotic disorder were used for the analyses described in this manuscript. Siblings and patients were compared as they are i) genetically more alike and ii) share more environmental variance than controls and patients [32]. The statistical power of case-sib designs in G×E studies may be greater than case-control designs when the correlation with E is low to moderate [32], [33], which is the case in the current study [1]. Furthermore, case-sibling designs are insensitive to population stratification bias and eliminate environmental/genetic confounders associated with unrelated controls [32]. Patients were recruited through representative clinicians whose caseload was screened for inclusion criteria. Siblings were sampled through participating patients. For 51 families, two or more participants took part in the study (2 participants (n = 46), 3 participants (n = 4), 4 participants (n = 1)). The composition of participants from one family was as follows: 1 sibling and 1 patient (n = 39), 2 siblings (n = 4), 2 patients (n = 3), 3 siblings (n = 1), 2 siblings and 1 patient (n = 3), 3 siblings and 1 patient (n = 1). Diagnoses were based on DSM-IV criteria, using the Comprehensive Assessment of Symptoms and History (CASH) interview [34]. Patient diagnoses were: schizophrenia (n = 69), brief psychotic disorder (n = 2), psychotic disorder not otherwise specified (n = 18). The CASH was additionally used to confirm the absence of non-affective psychosis in siblings [31]. Exclusion criteria were: I) brain injury with loss of consciousness >1 hour, II) meningitis/other neurological diseases, III) cardiac arrhythmia, IV) severe claustrophobia, V) metal corpora aliena (including intrauterine devices) VI) pregnancy. The ethics committee of the faculty of health, medicine and life science of Maastricht University approved the study. Written informed consent was obtained from every participant before participating in the study. All participants included in the study were able to give informed consent without the use of a legal representative or guardian.

### MRI

#### MRI acquisition

MRI scans were obtained on a 3T Siemens scanner (Erlangen, Germany). Acquisition parameters: Modified Driven Equilibrium Fourier Transform (MDEFT) sequence with 176 slices, isotropic voxel size of 1 mm, echo time = 2.4 ms, repetition time = 7.92 ms, inversion time = 910 ms, flip angle = 15°, total acquisition time = 12 m51 s. Magnetization Prepared Rapid Acquisition Gradient-Echo (MPRAGE) sequence with 192 slices, isotropic voxel size of 1 mm, echo time = 2.6 ms, repetition time = 2250 ms, inversion time =  900 ms, flip angle =  9°, total acquisition time = 7 m23 s. Matrix size was 256×256 and field of view was 256×256 mm^2^. The number of excitations was one. Two sequences were used because of a scanner update during data collection. The MPRAGE and MDEFT are similar, but to prevent any systematic bias, the total proportion of MPRAGE scans (approximately one third) was balanced between the groups [31].

#### MRI preprocessing and volume measures

Preprocessing was performed and structural volumetric measures were obtained using reconstruction and volumetric segmentation procedures from the freely available Freesurfer stable release version v5.0.0 (http://surfer.nmr.mgh.harvard.edu/), published in detail previously [11] and running on a Macintosh with OSX 10.6.4. In short, volumetric assessments of the left and right hippocampus were obtained for all participants using automatic classification procedures and labeling [35]. The FreeSurfer processing pipeline automatically assigns a neuroanatomical label, roughly corresponding to each voxel in an MRI volume (after partial volume correction), based on probabilistic information estimated from a manually labeled training set. The accurate labeling of subcortical structures is achieved through the use of both global and local information. The global information is based on an atlas that makes the labeling robust to contrast properties of the anatomical structures. Modeling the classification as a non-stationary anisotropic Markov random field incorporates local information. The introduction of non-stationary and anisotropy into the classical Markov random field model allows spatial relationships of anatomical classes to enter into the segmentation procedure. For instance, the probability that a voxel labeled “hippocampus” will have its inferior neighbor labeled as amygdala provides a strong set of spatial constraints. The output that FreeSurfer yields is rounded off to an integer number and therefore reflects the number of voxels in an area. The technique has previously been shown to be comparable in accuracy to manual labeling [35]. The segmentations were visually inspected for accuracy. A measure of intracranial volume (all voxels in a brain) was also generated, by adding up voxel counts for each area.

### Childhood Trauma

CT was assessed with the Dutch version of the Childhood Trauma Questionnaire Short Form (CTQ). The short CTQ consists of 25 items rated on a 5-point Likert scale (1 = never true to 5 =  very often true) inquiring about traumatic experiences in childhood. Five types of childhood maltreatment were assessed: emotional (mean = 1.6, sd = .77), physical (mean = 1.2, sd = .55) and sexual abuse (mean = 1.19, sd = .53), and emotional (mean = 2.1, sd = .84) and physical neglect (mean = 1.36, sd = .49). Five questions covered each type of trauma [36]. Calculating the mean of the 25 items resulted in a general measure of CT. CTQ data were missing for 9 participants (4.9%) (siblings = 4, patients = 5).

### Cognitive performance

Two neuropsychological tasks that rely on hippocampal functioning were included: the auditory verbal learning task (AVLT) and block design (BD) task, part of the Dutch version of the Wechsler Adult Intelligence Scale (WAIS IV). Delayed performance on the AVLT and BD performance assess recall capacity and spatial memory respectively, processes dependent on hippocampal integrity [28]–[30]. Delayed AVLT performance was defined as the number of words successfully recalled after a 15-minute interval (after a 15-word list was repeated three times). BD raw scores were calculated as the total amount of points after 14 trials. The task was ended prematurely when the participant scored no points on four consecutive trials. Depending on their speed, participants scored between zero and two points on each of the first six trials and between four to seven points on the remaining trials. AVLT and BD data were unavailable for 9,8% of the sample (16 siblings, 2 patients). AVLT performance was normally distributed. BD scores were exponentiated, after which they were normally distributed.

### Genes

The FKBP5 SNP selection was based on previous work [4] revealing an interaction between FKBP5 SNPs rs9296158, rs4713916, rs992105, rs3800373 and CT in the model of psychotic symptoms and cortisol levels later in life. BDNF SNP rs6265 (Val66Met) was selected on the basis of a reported interaction between rs6265 and CT in the context of psychotic symptoms later in life [3]. BDNF is a functional SNP (http://www.ncbi.nlm.nih.gov/gene/627) and selected FKBP5 SNPs have been consistently associated with functional outcomes [4], [18], [37], [38]. Genomic DNA was collected from blood. SNPs were determined by Sequenom (Hamburg, Germany), using the Sequenom MassARRAY iPLEX platform at the facilities of the manufacturer. The distribution of SNPs (among groups) is presented in Table S1. The selected SNPs were in Hardy-Weinberg equilibrium: rs9296158 (χ^2^ = 2.34, p = .18), rs4713916 (χ^2^ = .06, p = .83), rs992105 (χ^2^ = .03, p = .73), rs3800373 (χ^2^ = .35, p = .68) and rs6265 (χ^2^ = .87, p = .32). The distribution of genotypes among groups was not significantly different: rs9296158 (χ^2^ = .99, p = .61), rs4713916 (χ^2^ = .73, p = .69), rs992105 (χ^2^ = 2.93, p = .23), rs3800373 (χ^2^ = .2.88, p = .24) and rs6265 (BDNF) (χ^2^ = 1.14, p = 0.57). The linkage disequilibrium (LD) between FKBP5 SNPs has been described previously [4]. None of the selected FKBP5 SNPs were in 100% LD. No significant differences in demographic variables (table 1 for variables) on the basis of SNP genotype were observed (data available upon request).

**Table 1:** Sociodemographic variables for individuals with a diagnosis of psychotic disorder and healthy siblings.

	Siblings (n = 95)	Patients (n = 89)	Statistic	p
**Age, mean (SD)**	29.66 (8.79)	28.08(7.04)	−1.331	.18
**Gender, n (%)**			5.072	.02
male	50 (53%)	60 (69%)		
**Alcohol past 12 months (units/week), mean (SD)**	9.77 (17.1)	4.85 (8.97)	−2.281	.02
**Cannabis use past lifetime** 3 **(count), mean (SD)**	2.38 (2.61)	4.24 (3.24)	4.21	<.01*
**Other drug use lifetime** 3 **(count), mean (SD)**	1.38 (1.3)	2.7 (2.78)	4.151	<.01*
**Education** 4 **(finished), mean (SD)**	5.11 (2.04)	4.28 (1.98)		
**Childhood Trauma Questionnaire total score, mean (SD)**	6.88 (1.66)	8.16 (2.81)	3.731	<.01*
**Lifetime Haldol equivalents, mean (SD)**		6866.68 (6153.07)	−2.73	<.01*
**Scan type (MDEFT, ADNI), n (%)**			2	.16
MDEFT	59 (62%)	45 (52%)		

1 t-value

2 *Χ* ^2^-value

*p<.05

3 Cannabis and other drug values ranged from 1–8 (1 = 1–5 times, 2 = 6–9, 3 = 10–19, 4 = 20–39, 5 =  40 – 59, 6 = 60–79, 7 = 80–99, 8 = >100)

4 Education ranged from 1 (primary school) to master's degree (8)

MDEFT  =  Modified Driven Equilibrium Fourier Transform

MPRAGE  =  Magnetization Prepared Rapid Acquisition Gradient-Echo.

### Antipsychotic medication use

Antipsychotic medication use was determined by multiplying the number of days of antipsychotic medication use with the corresponding haloperidol equivalents and summing scores for all periods of antipsychotics use (including the exposure period between baseline assessment for the study and moment of MRI scan), using the published converting formulas for antipsychotic medication dose equivalents described by Andreasen and colleagues' [39]. 11 patients used antidepressants.

### Analyses

For hippocampal volume, the number of voxels in the left and right hippocampus was used as continuous dependent variable, an indication of the structure's size. For cognitive performance, we used delayed AVLT and (exponentiated) BD performance as a continuous measure. The effect of group (patients vs. siblings; categorical), genotype (BDNF/FKBP5 genotype; categorical) and CT (total CTQ score; continuous) on hippocampal volume/cognitive performance was first explored separately. Association analyses between hippocampal volume and antipsychotic medication use were also performed. Next, we investigated the role of BDNF/FKBP5 genotype in the association between CT and hippocampal volume/cognitive performance in adulthood in the whole sample (hippocampal volume/cognitive performance  =  genotype*CT), guided by recent mixed results on the role of the BDNF gene in the association between childhood adversity and hippocampal volume [22], [23]. We then investigated whether the variable “group” (patients, siblings) moderated the association between CT and hippocampal volume/cognitive performance. Finally, it was investigated whether “group” moderated the effect of BDNF/FKBP5 genotype in the association between CT and hippocampal volume/cognitive performance (hippocampal volume/cognitive performance  =  genotype*CT*group). For this analysis, group was added as an independent variable (section 3.3). Given the low number of homozygous minor allele carriers (Table S1), risk allele carriers were grouped for genetic analyses [major allele homozygotes = [1], minor allele heterozygotes and homozygotes = [2]).

### Statistical model, covariates and software

Demographic analyses were performed using linear regression and chi-square tests (REGRESS and TAB command in STATA 11.0 respectively). All remaining regression analyses were mixed models and were performed using the XTMIXED command in Stata 11.0 (StataCorp LP, College Station, TX, 2013) with family ID as random effect [31]. Cannabis use (scale, lifetime) [40], [41], other drug use (scale, lifetime) [42] and alcohol consumption [43] were assessed using the Composite International Diagnostic Interview (WHO, 1990). Cannabis use and other drug use were scored on 8-point scales (1 = 1–5 times, 2 = 6–9, 3 = 10–19, 4 = 20–39, 5 =  40 – 59, 6 = 60–79, 7 = 80–99, 8 = >100)). These two scales were averaged per person to create a “drug use” covariate.

Volumetric analyses were corrected for intracranial volume, age, gender [22], [23], educational level (8-point scale that ranged from primary school (1) to master's degree (8)), drug use and alcohol consumption (drug use and alcohol consumption hereafter: “substance use”). AVLT and BD analyses were corrected for age, gender and substance use. AVLT and BD analyses were not corrected for educational level because of their high collinearity. The Bonferroni-corrected threshold for genotype analyses was p = .005 (10 tests; 5 SNPs × left and right hippocampal volume/AVLT and BD performance). Covariate × E and covariate × G were included in every G×E analysis [24]. Covariate × E and covariate × G were included in G and E interactions respectively. Model fit tests, using a two-tailed likelihood ratio test, were performed to select the optimal statistical model and demonstrate the effect of relevant covariates on model fit. Although the scantype (mdeft/adni) was equally distributed among groups, all analyses were repeated with scantype as covariate. Adding scantype as a covariate did not affect the (non-)significance of any of the results. Adding antidepressant use as a covariate also did not affect the outcomes. CT analyses were repeated using a dichotomized variable (dichotomized at the 80th percentile of the trauma scores of controls; see Heins et al[1]), but did this not affect the results.

## Results

### Demographics and main effects

#### Demographics

Demographics are shown in table 1. Patients differed from siblings on education, cannabis use, use of other drugs (patients>siblings) and alcohol consumption (patients<siblings). Education, cannabis and other drug (as “drug use”) were therefore included in all relevant analyses (next to age, gender and intracranial volume) (see also section 2.8).

#### Model choice

Given that groups differed on some sociodemographic variables (e.g. drug use) (table 1), model fit tests were performed to determine the optimal statistical model. Model fit was assessed using likelihood ratio tests (Likelihood ratio  =  −2 ln(L(model1)/L(model2))  =  2(ll(model2)-ll(model1))). This ratio test yielded a chi-square statistic and p-value, with significant p-values indicating that model 2 (special case of model 1) fits the data better. For both hippocampal volume and cognitive performance, a model including substance use (in addition to intracranial volume (volumetric analyses only), age and gender) provided a significantly better model fit than a model without substance use at all levels (Table S2). More parsimonious models (i.e. leaving one of the substance use variables out of the model) did not provide systematic improvements in model fit at all levels (data not shown). Therefore, a model including all substance use items (alcohol, drug use), added as separate variables, was used for all analyses reported below.

#### Main effects

A strong group difference was found in left hippocampal volume (patients<siblings) (table 2), which remained significant after controlling for substance use. No significant association between SNP genotype (BDNF/FKBP5) and hippocampal volume was observed (model including substance use: p-values ranging from.22 to.93, data not shown), and the same held true for the association between CT and hippocampal volume (table 2). Siblings performed better on the delayed AVLT task, only when substance use was included. Siblings only performed better on the BD task than patients when substance use included in the model (table 2). No association between BDNF/FKBP5 genotype and delayed AVLT (model including substance use: p-values ranged from.07 to.66, data not shown) or BD performance (model including substance use: p-values ranged from.19 to.61 data not shown) was observed, even when omitting substance use.

**Table 2:** Various main-effect associations with and without substance use included in the model.

Hypothesis	Substance use included				Substance use excluded			
	B	Z	95% CI	P	B	Z	95% CI	P
L HC = group	−181.86	−2.72	−313.08 to –50.64	<.01*	−161.41	−2.76	−276.13 to –46.68	<.01*
R HC = group	−116.95	−1.73	−249.19 to 15.3	.08	−71.42	−1.12	−196.34 to 53.5	.26
L HC = CT	1.28	.08	−31.91 to 34.47	.94	−15.74	−1.06	−44.77 to 13.28	.29
R HC = CT	14.2	.8	−20.41 to 48.81	.42	−1.31	−.08	−33.04 to 30.41	.94
AVLT = group	−1.23	−2.65	−2.14 to –.32	<.01*	−.99	−1.96	−1.98 to 0	.05
BD = group	−523.36	−2.39	−952.33 to –94.39	.02*	−470.57	−2.59	−827.29 to –113.85	.01*
AVLT = CT	−.11	−.93	−.33 to.12	.35	−.13	−1.3	−.32 to.07	.2
BD = CT	−120.47	−2.29	−223.47 to –17.47	.02*	−107.3	−2.48	−192.01 to –22.59	<.01*
Drug use = CT	N/A	N/A	N/A	N/A	.38	5.07	.24 to.53	<.01*
Alcohol = CT	−.89	−1.57	−2 to.23	.12	−.49	−.94	−1.51 to.53	.35

*p<.05

HC = hippocampus.

Higher levels of CT were negatively associated with BD performance, even when substance use was excluded from the model (table 2). CT was not associated with AVLT performance. CT was positively associated with drug use, but not with alcohol consumption (table 1). Substance use was not associated with genotype (data not shown).

#### Medication

Best-estimates of lifetime cumulative antipsychotic medication use were not associated with left or right hippocampal volume in patients (left: B = <.01, Z = ..69, 95% CI = −.01 –.02, p = .49; right: B<.01, Z = .36, 95% CI = −.012 –.017, p = .72). No association between lifetime cumulative antipsychotic medication and delayed AVLT (B<.01, Z = .68, 95% CI = 0 – <.01, p = .49) or BD performance (B<−.02, Z = −.86, 95% CI = −.07 –.03, p = .39) was observed.

### The role of group/genotype in the association between childhood trauma and hippocampal volume/cognitive performance

#### Hippocampal volume

Convincing CT by group interactions in the model of left and right hippocampal volume were not observed (left: B =  −75.36, Z = −1.9, 95% CI =  −152.91 − 2.18, p = .06; right: B = −67.17, Z = −1.82, 95% CI =  −145.16 – 19.41, p = .13), even when substance use was included (left: B =  −65.64, Z = −1.84, 95% CI =  −135.59 – 4.3, p = .07; right: B =  −72.88, Z = −1.86, 95% CI = −149.83 – 4.08, p = .06). Variations within the selected FKBP5 SNPs, rs9296158, rs4713916, rs992105, rs3800373 and BDNF did not significantly interact with CT to influence hippocampal volume (table 3: model including substance use), regardless of the addition of substance use to the model.

**Table 3:** FKBP5 and BDNF SNP genotype do not affect the association between childhood trauma and hippocampal volume (no group).

SNP	Area	B	95% CI	Z	p
rs9296158	L HC	61.43	−57.31 to 180.17	1.01	.31
	R HC	62.26	−58.1 to 182.62	1.01	.31
rs4713916	L HC	47.73	−38 to 133.45	1.09	.28
	R HC	49.33	−37.42 to 136.08	1.11	.27
rs992105	L HC	48.14	−49.07 to 145.36	.97	.33
	R HC	63.78	−35.24 to 162.8	1.26	.21
rs1360780	L HC	−7.72	−95.48 to 80.04	−.17	.86
	R HC	13.74	−76.14 to 103.62	.3	.77
BDNF (rs6265)	L HC	25.82	−102.41 to 154.05	.39	.69
	R HC	36.79	−93.1 to 166.67	.56	.58

Group coding: sibling = [0], patient = [1]

Genotype coding: dichotomous, major allele homozygotes vs. minor allele hetero- and homozygotes (see section 2.7). Childhood trauma score: continuous. P(corrected) = .005. Covariates: intracranial volume, age, gender, education, substance use. Random factor: family id. HC = hippocampus.

#### Cognitive performance

CT and group did not interact in the model of delayed AVLT/BD performance (model including substance use: AVLT: B = −.14, Z = −.52, 95% CI =  −.66 –.38, p = .6; BD: B =  −49.5, Z = −.39, 95% CI =  −299.57 – 200.57, p = .7), regardless of substance use. Variations within rs9296158, rs4713916, rs992105, rs3800373 and BDNF did not interact with CT in the model of AVLT/BD performance (table 4: model including substance use), regardless of substance use.

**Table 4:** FKBP5 and BDNF SNP genotype do not affect the association between childhood trauma and AVLT and BD performance (no group).

SNP	Performance	B	95% CI	Z	p
rs9296158	AVLT	.38	−.33 to 1.08	1.05	.29
	BD	89.42	−240.73 to 419.56	.53	.6
rs4713916	AVLT	.16	−.38 to.71	.6	.55
	BD	70.08	−190.91 to 331.07	.53	.6
rs992105	AVLT	.54	−.1 to 1.18	1.64	.1
	BD	428.55	128.17 to 728.93	2.8	<.01
rs1360780	AVLT	.27	−.26 to.8	.99	.32
	BD	128.03	−130.13 to 386.19	.97	.33
BDNF (rs6265)	AVLT	−.14	−.43 to –.7	.48	.63
	BD	29.63	−238.56 to 297.81	.22	.83

Group coding: sibling = [0], patient = [1]

Genotype coding: dichotomous, major allele homozygotes vs. minor allele hetero- and homozygotes (see section 2.7). Childhood trauma score: continuous. P(corrected) = .005. Covariates: age, gender, substance use. Random factor: family id.

### The moderating role of BDNF/FKBP5 SNP genotype in the association between childhood trauma and hippocampal volume/cognitive performance

#### Hippocampal volume

No evidence for group X FKBP5 X CT interactions was observed for hippocampal volume (table 5: model including substance use) when substance use was added to the model. When substance use was omitted, there was a trend-significant BDNF × CT × group interaction for left hippocampal volume at the p(corrected) threshold of.005 (left: B =  215.47, Z = 2.35, 95% CI = 35.55 – 395.39, p = .02; right: B =  143.83, Z = 1.47, 95% CI =  −48.57 – 336.23, p = .14). This tentative interaction indicated the following: CT decreased left hippocampal volume in sibling Met-allele carriers (relative to sibling Val homozygotes), while CT increased left hippocampal volume in patient Met-allele carriers (relative to patient Val homozygotes).

**Table 5:** No significant effect of group (sibling, patient) on FKBP5 and BDNF SNP genotype x childhood trauma interactions in the model of hippocampal volume.

SNP	Area	B	95% CI	Z	P
rs9296158	L HC	161.47	−60.8 to 383.74	1.42	.16
	R HC	204.93	−24.53 to 434.39	1.75	.08
rs4713916	L HC	12.77	−175.75 to 201.3	.13	.89
	R HC	−1.39	−197.62 to 194.84	−.01	.99
rs992105	L HC	142.62	−69.96 to 355.1	1.31	.2
	R HC	95.29	−126.47 to 317.05	.84	.4
rs1360780	L HC	44	−157.1 to 245.09	.43	.67
	R HC	50.82	−154.6 to 256.25	.48	.63
BDNF (rs6265)	L HC	141.06	−93.56 to 375.68	1.18	.24
	R HC	37.82	−207.44 to 283.08	.3	.76

Group coding: sibling = [0], patient = [1]

Genotype coding: dichotomous, major allele homozygotes vs. minor allele hetero- and homozygotes (see section 2.7). Childhood trauma score: continuous. P(corrected) = .005. Covariates: intracranial volume, age, gender, education, substance use. Random factor: family id. HC = hippocampus.

#### Cognitive performance

No evidence for group × FKBP5/BDNF × CT interactions in the model of delayed AVLT/BD performance was observed (table 6: model including substance use).

**Table 6:** No significant effect of group (sibling, patient) on BDNF SNP genotype x childhood trauma interactions in the model of AVLT and BD performance.

SNP	Performance	B	95% CI	Z	P
rs9296158	AVLT	.07	−1.27 to 1.41	.1	.92
	BD	163.47	−496.54 to 823.48	.49	.63
rs4713916	AVLT	−.56	−1.73 to.61	−.94	.35
	BD	53.65	−535.89 to 643.19	.18	.86
rs992105	AVLT	−.24	−1.55 to 1.06	−.37	71
	BD	−108.24	−765.49 to 549	−.32	.75
rs1360780	AVLT	−.21	−1.43 to 1.01	−.33	.74
	BD	77.29	−525.94 to 680.52	.25	.8
BDNF (rs6265)	AVLT	−1.35	−2.6 to.11	−2.13	.03
	BD	−134.33	−774.4 to 505.75	−.41	.68

Group coding: sibling = [0], patient = [1]

Genotype coding: dichotomous, major allele homozygotes vs. minor allele hetero- and homozygotes (see section 2.7). Childhood trauma score: continuous. P(corrected) = .005. Covariates: age, gender, substance use. Random factor: family id.

## Discussion

We investigated whether FKBP5 and BDNF genotype moderated the association between CT and two proxies of hippocampal integrity differently in individuals with a diagnosis of a psychotic disorder, compared to healthy siblings. We presented evidence that hippocampal volume and cognitive performance (on a delayed AVLT and BD task) were not affected by a BDNF/FKBP5, CT and group status interaction, at least, when adequately controlling for the undesired influence of drug use and alcohol consumption.

### The moderating effects of genes on the association between childhood trauma and hippocampal volume

BDNF and FKBP5 genotype did not moderate the association between CT and hippocampal volume assessed later in life, regardless of group status. This is the first investigation looking at FKBP5 genotype by CT interactions in the model of hippocampal volume. Our BDNF findings parallel those of a recent study [23], who also did not observe a significant BDNF genotype by childhood adversity interaction on, among others, hippocampal volume in a large sample of healthy volunteers screened for a past of illicit drug and other substance use. Similarly, no differences in levels of hippocampal BDNF gene expression were observed in maltreated and control rats in an animal model of childhood adversity, yet differences in the prefrontal cortex (PFC) were present [19]. These findings seemingly disagree with those of Carballedo and colleagues [22], who observed an association between BDNF genotype and hippocampal volume when combining their sample of patients with depressive disorder (MDD) and healthy controls: for a given amount of exposure to childhood adversity, Met-allele carriers had a smaller hippocampal volume than Val homozygotes. The results in the present study suggest the absence of such interactions when alcohol consumption and drug use are included in the model, factors that have been demonstrated to affect brain structure [40]–[43]. Differences in sample characteristics, studied populations (MDD vs. psychotic disorder), but also relevant inclusion of covariates and their E and G interactions [24] could explain the discrepancy in results.

No main effects of BDNF genotype on hippocampal volume were observed, in agreement with a recent inconclusive meta-analysis [44], although left hippocampal volume was dependent on group status. A trend-significant group by CT by BDNF interaction in the model of left hippocampal volume was largely due to the confounding effects of substance use and disappeared after adequate inclusion of covariates and their G/E interactions. Our results indicate that assessments of hippocampal volume can be misrepresented when not taking into account the effect of substance use. Importantly, the provocative results we present could partially explain why published reports on the effect of stressful events during childhood and hippocampal volume later in life have been mixed (positive: [10], [11], inconclusive: [20], [21]). Moreover, they could justify the inclusion of relevant covariates and careful evaluation of statistical models (e.g. model fit tests, covariate times G/E interactions) in an attempt to discern between true association and confounding.

### Gene by childhood trauma interactions on cognitive and affective domains

In concordance with our volumetric results, CT by BDNF/FKBP5 genotype interactions, with or without the inclusion of group status, were not observed in the model of delayed AVLT and BD performance. The presented results are the first to indicate that FKBP5 genotype does not affect the association between CT (or CT by group status interactions) and hippocampal-dependent cognition. Although BDNF did not influence the association between CT interactions and cognition, BDNF genotype has been shown to impact on the association between sexual abuse and cognition [45]. Importantly, in the group of individuals without sexual abuse, a type of abuse rare in our sample (section 2.3), cognition was not dependent on BDNF genotype [45], which is in line with the presented results. There was no main effect of FKBP5 genotype variation on delayed AVLT and BD performance, as was the case for BDNF.

Although studies investigating BDNF/FKBP5 by CT interactions in the model of cognition are scarce, and associations between BDNF genotype on cognition are modestly strong at best [46], the association between CT and outcome measures in affective domains has been reported to be dependent on FKBP5 and BDNF genotype. These studies have shown that the experience of CT in BDNF Met allele carriers is associated with increased levels of psychotic symptoms [3], could be related to depressive symptoms [47] and increase the impact of life events on bipolar illness [48], compared to Val allele carriers. Similarly, FKBP5 minor alleles seem to amplify the negative effects of CT on depression [49], threat-related brain activity [50], psychotic symptoms and cortisol levels later in life [50]. Interestingly, a study that found mixed effects of FKBP5 genotype on multiple indexes of hippocampal structure, did find a marked association between FKBP5 genotype and threat-related hippocampal activity, again, with the minority allele associated with heightened activity [51].

Previously cited studies demonstrate a potential role for BDNF and FKBP5 minority alleles in the association between CT and changes in affective domains later in life. The absence of consistent association with cognition, including those presented in the current manuscript, could suggest that BDNF/FKBP5 genoype by CT interactions impact on an affective, rather than cognitive, pathway towards psychopathology later in life [52]. The absence of associations with hippocampal volume, in combination with the presence of other, widespread, changes in the stress network (e.g. cortisol [4], threat-related brain activity [51], could suggest that these interactions are more likely to affect a larger network, than to impact on one brain structure in particular.

### Strengths and limitations

The consistent absence of any FKBP5/BDNF genotype × CT interactions could have been the result of a power problem (e.g. CT by group by BDNF interaction in the model of delayed AVLT). Although similar in sample size to other published work [22], complex interactions such as the one reported under section 3.3 are generally investigated in much larger samples. Although it is unlikely that the study was underpowered for main effects and two-way interactions, the absence of three-way interactions should be interpreted with caution and replicated in larger samples. Moreover, the data presented in this manuscript are cross-sectional. Conclusions drawn from these results do not imply causality and can not answer questions with regard to the temporal association between CT, hippocampal volume and cognition. Furthermore, CT was retrospectively assessed in this study, which could have lead to over- or underestimations of the actual prevalence and impact of CT. It is also unlikely that the genes investigated in the current study are solely responsible for G×E in the context of CT and psychosis. Although genes were a-priori selected, based on previous evidence [3], [4], polygenic risk scores or the incorporation of multiple genes associated with the human stress response might have uncovered more subtle G×E interactions that were not observed in the current study.

In order to provide an accurate reflection of hippocampal integrity, two proxies were investigated. The degree of consistency between those results may have benefitted the accuracy of the conclusions. Furthermore, including influential sample characteristics (genetic, environmental, demographic and lifestyle factors) and the use of appropriate covariates (times G/E) [24] may have attributed to the validity of the results. Finally, we compared individuals at above average genetic risk, siblings, with a patient sample. These two groups may be genetically more alike, share more environmental variance and therefore more easily compared than controls and patients, who are environmentally and genetically less alike [32].

Data presented in this manuscript is available to collaborators upon request.

## Supporting Information

Table S1 BDNF/FKBP5 genotype distribution for siblings and patients.
(DOC)

Table S2 Model fit tests comparing a statistical model including substance use to a model without substance use.
(DOC)
