# Supplementary material for: Brain-Derived Neurotrophic Factor/FK506-Binding Protein 5 Genotype by Childhood Trauma Interactions Do Not Impact on Hippocampal Volume and Cognitive Performance
Source: PLoS One. 2014 Mar 21;9(3):e92722. doi: 10.1371/journal.pone.0092722 (PMC3962453; doi:10.1371/journal.pone.0092722)
Supplement: Table S1 — BDNF/FKBP5 genotype distribution for siblings and patients. (DOC) [file pone.0092722.s001.doc]

**Table S1** BDNF/FKBP5 genotype distribution for siblings and patients

| **rs9296158** | **siblings** | **patients** | **Total** |
| --- | --- | --- | --- |
| **G/G** | 42 | 34 | 76 |
| **A/G** | 29 | 33 | 62 |
| **A/A** | 3 | 3 | 6 |

| **rs4713917** | **siblings** | **patients** | **Total** |
| --- | --- | --- | --- |
| **G/G** | 54 | 46 | 100 |
| **A/G** | 29 | 31 | 60 |
| **A/A** | 6 | 4 | 10 |

| **rs992105** | **siblings** | **patients** | **Total** |
| --- | --- | --- | --- |
| **A/A** | 77 | 57 | 134 |
| **A/T** | 15 | 22 | 37 |
| **T/T** | 2 | 1 | 3 |

| **rs3800373** | **siblings** | **patients** | **Total** |
| --- | --- | --- | --- |
| **C/C** | 54 | 40 | 94 |
| **C/T** | 29 | 37 | 66 |
| **T/T** | 5 | 4 | 9 |

| **BDNF (rs6265)** | **siblings** | **patients** | **Total** |
| --- | --- | --- | --- |
| **Val/Val** | 50 | 44 | 94 |
| **Val/Met** | 20 | 24 | 44 |
| **Met/Met** | 5 | 3 | 8 |
