# Supplementary material for: Brain-Derived Neurotrophic Factor/FK506-Binding Protein 5 Genotype by Childhood Trauma Interactions Do Not Impact on Hippocampal Volume and Cognitive Performance
Source: PLoS One. 2014 Mar 21;9(3):e92722. doi: 10.1371/journal.pone.0092722 (PMC3962453; doi:10.1371/journal.pone.0092722)
Supplement: Table S2 — Model fit tests comparing a statistical model including substance use to a model without substance use. (DOC) [file pone.0092722.s002.doc]

**Table S2** Model fit tests comparing a statistical model including substance use to a model without substance use

| **Outcome measure** | **Model** | **Covariates** | **LL** | Χ^2^ | **p** |
| --- | --- | --- | --- | --- | --- |
| **L Hippocampus** | Main effect group | Intracranial volume, gender, age, education | -1159.66 | 349.66 | <.01 |
|  | Main effect group | Intracranial volume, gender, age, education addictive substances | -984.83 |
|  | Group*childhood trauma^1^ | Intracranial volume, gender, age, education | -1079.9 | 365.55 | <.01 |
|  | Group*childhood trauma^1^ | Intracranial volume, gender, age, education, addictive substances | -897.13 |
|  | Group*childhood trauma*rs4713916^2^ | Intracranial volume, gender, age, education | -960.93 | 367.05 | <.01 |
|  | Group*childhood trauma*rs4713916^2^ | Intracranial volume, gender, age, education, addictive substances | -777.4 |
| **R Hippocampus** | Main effect group | Intracranial volume, gender, age, education | -1174.85 | 361.07 | <.01 |
|  | Main effect group | Intracranial volume, gender, age, education, addictive substances | -994.31 |
|  | Group*childhood trauma^1^ | Intracranial volume, gender, age, education | -1093.67 | 375.33 | <.01 |
|  | Group*childhood trauma^1^ | Intracranial volume, gender, age, education, addictive substances | -906 |
|  | Group*childhood trauma*BDNF^2^ | Intracranial volume, gender, age, education | -842.4 | 347.18 | <.01 |
|  | Group*childhood trauma*BDNF^2^ | Intracranial volume, gender, age, education, addictive substances | -668.8 |
| **AVLT** | Main effect group | Gender, age | -399.99 | 107.98 | <.01 |
|  | Main effect group | Gender, age, addictive substances | -346 |
|  | Group*childhood trauma^1^ | Gender, age | -382.45 | 105.21 | <.01 |
|  | Group*childhood trauma^1^ | Gender, age, addictive substances | -329.85 |
|  | Group*childhood trauma*rs9296158^2^ | Gender, age | -295.49 | 84.28 | <.01 |
|  | Group*childhood trauma*rs9296158^2^ | Gender, age, addictive substances | -253.35 |
| **BD** | Main effect group | Gender, age | -1365.84 | 402.12 | <.01 |
|  | Main effect group | Gender, age, addictive substances | -1164.78 |
|  | Group*childhood trauma^1^ | Gender, age | -1276.87 | 416.94 | <.01 |
|  | Group*childhood trauma^1^ | Gender, age, addictive substances | -1068.34 |
|  | Group*childhood trauma*rs992105^2^ | Gender, age | -1126.14 | 427.3 | <.01 |
|  | Group*childhood trauma*rs992105^2^ | Gender, age, addictive substances | -912.49 |

Model fit was assessed using likelihood ratio tests (Likelihood ratio = -2 ln(L(model1)/L(model2)) = 2(ll(model2)-ll(model1))).

LL= log-restricted likelihood

^1^difference in degrees of freedom=2

^2^contains covariate*E terms; difference in degrees of freedom=4

^3^contains covariate*G and covariate*E terms; difference in degree of freedom=6
P-value <.05 indicates a better model fit for the second complicated model.
Substance use: drug use (mean scale, lifetime), alcohol consumption (units/week, past 12 months), SNPs mentioned in table indicate the least significant improved model fit, compared to other SNPs in the same analyses.
AVLT = Auditory Verbal Learning Task (delayed performance)
BD = Block Design
